# MicroRNA profiling of primary pulmonary enteric adenocarcinoma in members from the same family reveals some similarities to pancreatic adenocarcinoma—a step towards personalized therapy

**DOI:** 10.1186/s13148-015-0162-5

**Published:** 2015-12-16

**Authors:** Ingrid Garajová, Niccola Funel, Michelangelo Fiorentino, Valentina Agostini, Manuela Ferracin, Massimo Negrini, Giovanni Luca Frassineti, Giampaolo Gavelli, Adam Enver Frampton, Guido Biasco, Elisa Giovannetti

**Affiliations:** Department of Medical Oncology, VU University Medical Center, Cancer Center Amsterdam, CCA room 1.52, De Boelelaan 1117, 1081 HV Amsterdam, The Netherlands; Department of Experimental, Diagnostic and Speciality Medicine, Sant’Orsola-Malpighi Hospital, University of Bologna, Via Massarenti 9, 40138 Bologna, Italy; Division of General and Transplant Surgery, Pisa University Hospital, Via Paradisa 2, 56124 Pisa, Italy; Cancer Pharmacology Lab, AIRC Start-Up Unit, University of Pisa, Lungarno Pacinotti 43, 56126 Pisa, Italy; Department of Pathology, F. Addari Institute, S.Orsola Malpighi Hospital, University of Bologna, Viale Ercolani 4/2, 40138 Bologna, Italy; Department of Morphology, Surgery and Experimental Medicine, University of Ferrara, Via Borsari 46, 44121 Ferrara, Italy; Laboratory for Technologies of Advanced Therapies (LTTA), University of Ferrara, Via Fossato di Mortara 70, 44121 Ferrara, Italy; Department of Medical Oncology, Istituto Scientifico Romagnolo per lo Studio e la Cura dei Tumori (IRST) IRCCS, via Piero Maroncelli 40, 47014 Meldola, Italy; Department of Radiology, Istituto Scientifico Romagnolo per lo Studio e la Cura dei Tumori (IRST) IRCCS, via Piero Maroncelli 40, 47014 Meldola, Italy; HPB Surgical Unit, Department of Surgery & Cancer, Hammersmith Hospital Campus, Imperial College, Du Cane Road, London, W12 0HS UK; Cancer Pharmacology Lab, Start-Up Unit, University of Pisa, via Paradisa 2, 56124 Pisa, Italy

**Keywords:** Pulmonary adenocarcinoma, Enteric, Intestinal, Immunohistochemistry, PEAC

## Abstract

**Background:**

Primary pulmonary enteric adenocarcinoma (PEAC) is defined as a pulmonary adenocarcinoma with a predominant component of intestinal differentiation and tumor cells positive for at least one intestinal marker. The aim of the present study was the molecular and histological characterization of a PEAC from a patient with two other family members affected by similar lung tumors, which has never been reported before.

**Findings:**

We evaluated the molecular characteristics of the proband’s PEAC by using a previously validated 47-microRNA (miRNA) cancer-specific array and a predictive method to estimate tissue-of-origin probabilities. Immunohistochemical (IHC) staining for thyroid transcription factor (TTF-1), napsin A, caudal-related homeobox 2 (CDX2), cytokeratins, and mucins, as well as mutational analyses for epidermal growth factor receptor (EGFR), Kirsten rat sarcoma viral oncogene homolog (KRAS), and anaplastic lymphoma kinase (ALK) were performed on formalin-fixed, paraffin-embedded (FFPE) tissues.

The occurrence of PEAC in two family members was associated with similar clinicopathological features (age at diagnosis, smoking habit, tumor localization, multiple colonic polyps), histologic findings (TTF-1 negativity and CDX2 positivity), and genetic findings (KRAS (Gly12Asp) mutation, but no EGFR/ALK aberrations). miRNA profiling revealed similarities with non-small cell lung cancer (NSCLC; 75.98 %) and some overlap with pancreatic ductal adenocarcinoma (PDAC; 23.34 %), but not with colorectal cancer (CRC; less than 0.5 %). Notably, these PEACs share key PDAC-associated miRNAs associated with tumor aggressiveness (miR-31*/-126*/-506/-508-3p/-514).

**Conclusions:**

We describe for the first time PEAC in members from the same family, associated with similar clinical and genetic features. miRNA profiling of the PEAC resembled a NSCLC signature, with partial overlap to a PDAC pattern. This could explain its aggressive behavior and therefore help to guide future tailored-therapeutic approaches.

**Electronic supplementary material:**

The online version of this article (doi:10.1186/s13148-015-0162-5) contains supplementary material, which is available to authorized users.

## Findings

### Introduction

Primary pulmonary enteric adenocarcinoma (PEAC) is defined as a pulmonary adenocarcinoma with a predominant component (>50 %) of intestinal differentiation and with tumor cells positive for at least one intestinal marker such as caudal-related homeobox 2 (CDX2), CK20, or MUC2 [1]. It was first described in 1991 [2], and a total of 31 cases have been reported in the literature [2–13]. In the International Multidisciplinary Classification of Lung Adenocarcinoma (2011), PEAC was classified as a rare variant of invasive adenocarcinoma [1]. The overlap between PEAC and metastases from other tumors (such as colorectal cancer (CRC)) represents an important diagnostic problem [2–13]. Several studies have demonstrated the ability of microRNA (miRNA) profiling for the identification of the tissue of origin for ambiguous cancers, assisting in the diagnosis of carcinomas of unknown origin [14, 15].

In the present study, we performed miRNA profiling in conjunction with immunohistochemical (IHC) and genetic analyses on a formalin-fixed, paraffin-embedded (FFPE) tissue from a patient affected by PEAC. Notably, this patient had a sister and a brother who were also affected by PEAC, and we describe the main clinicopathological findings in these family members.

## Materials and methods

### Patients

The proband was a 68-year-old male, ex-smoker, with no history of malignancy or relevant comorbidities. Computed tomography (CT) and positron emission tomography ([^18^F]FDG-PET) showed a 3.5-cm nodule in the right lower lobe with high glucose uptake. Lobectomy revealed a stage-IIA non-small cell lung cancer (NSCLC) with PEAC histology. Colonoscopy showed multiple benign polyps, but excluded primary CRC. Post-operative CT and [^18^F]FDG-PET revealed a small (8 mm) osteolytic area in the left hip bone. Considering the predominant component of intestinal differentiation, the patient was treated with XELOX (capecitabine + oxaliplatin) and a bisphosphonate, though with a very short CT scan re-evaluation, which after 2 cycles of chemotherapy showed progression of the bone lesion (2 cm). Consequently, the patient underwent surgical removal of this unique bone lesion, which was confirmed histologically as a PEAC metastasis. After 4 months, the patient relapsed with multiple bone metastases and underwent 4 cycles of carboplatin + pemetrexed which did not arrest tumor progression. Subsequent chemotherapy with docetaxel resulted in disease stability after 2 cycles.

Similarly, the 71-year-old proband’s sister underwent lung lobectomy revealing a stage-IB PEAC (Table 1), which progressed in 12 months with lung and adrenal gland metastases, but after 6 cycles of carboplatin + pemetrexed, followed by pemetrexed alone, became stable. Another brother (72 years old) presented with an advanced stage lung tumor (pleural effusion and multiple lytic bone metastases) and succumbed 1 month after diagnosis.

**Table 1:** Demographic and disease characteristics of two patients affected by PEAC within one family. The familial aggregation of PEAC was associated with similar clinicopathological features (age at diagnosis, smoking habit, tumor localization, multiple colon polyps), histologic findings (IHC staining negative for TTF-1 and positive for CDX2), and genetic findings (KRAS(Gly12Asp) mutation but no EGFR/ALK aberrations)

Characteristic	Proband		His sister
General			
Age at diagnosis (years)	68		71
Smoking habit	Yes		Yes
Primary localization	Lung: right/lower lobe		Lung: right/lower lobe
Colonoscopy	Multiple benign polyps (*N* < 10), diverticulosis		Multiple benign polyps (*N* < 10), diverticulosis
Radical surgery (R0)	Yes		Yes
Metastases/DFS (months)	Bone/1.5		Lung, adrenal glands/12
Histological findings	Primary tumor	Bone metastasis	
Histology	PEAC	PEAC	PEAC
Stage (pTNM)	pT2apN1		pT2apN0
IHC analyses			
TTF-1	NEG	NEG	NEG
Napsin A	NEG	NA	NA
CK7	POS	NA	NEG
CK20	NEG	NA	POS
CDX2	POS	POS	POS
MUC1	POS	NA	NA
MUC2	NEG	NA	NA
MUC5AC	POS	NA	NA
MUC6	NEG	NA	NA
Microsatellite instability	MSS phenotype	NA	NA
Genetics			
EGFR	Wild type	NA	Wild type
KRAS	Gly12Asp mutation	Gly12Asp mutation	Gly12Asp mutation
ALK	Wild type	NA	Wild type

*Abbreviations*: *ALK* anaplastic lymphoma kinase, *CDX2* caudal-related homeobox 2, *CK7* cytokeratonin-7, *CK20* cytokeratonin-20, *DFS* disease-free survival, *EGFR* epidermal growth factor receptor, *IHC* immunohistochemistry, *KRAS* Kirsten rat sarcoma viral oncogene homolog, *MUC1* mucin 1, *MUC2* mucin 2, *MUC5AC* mucin 5A, *MUC6* mucin 6, *NA* non available, *NEG* negative, *PEAC* primary pulmonary enteric adenocarcinoma, *POS* positive, *TTF-1* thyroid transcription factor, *pTNM* pathological tumor-node-metastasis

#### IHC and genetic and microarray analyses

The IHC analysis evaluated thyroid transcription factor (TTF-1), napsin A, cytokeratins (CK7/CK20), CDX-2, and mucins (MUC1/MUC2/MUC5AC/MUC6). For the genetic analyses, the neoplastic area was macro-dissected using a parallel hematoxylin and eosin (H&E) slide as a reference, with subsequent DNA/RNA isolation from samples with >80 % tumor infiltration. Epidermal growth factor receptor (EGFR) and Kirsten rat sarcoma viral oncogene homolog (KRAS) analyses were performed by PCR and direct sequencing, respectively. Anaplastic lymphoma kinase (ALK) status was studied by fluorescence in situ hybridization. Microsatellite instability (MSI) was evaluated by multiplex amplification and sequencing.

miRNA profiling was performed using the Agilent Human miRNA microarray v.2 (#G4470B; Agilent Technologies, Santa Clara, CA, USA). The Feature Extraction 10.7 and GeneSpring GX13 software were used to analyze the microarray raw, and we applied a previously validated method to estimate tissue-of-origin probabilities [15]. Manhattan correlation was used as a measure of similarity.

No samples for further pathological and genetic analyses were available from the proband’s sister, while cytological specimens of the proband’s brother were available to evaluate TTF-1, CDX-2, CK7, CK20, and for PCR analysis of selected miRNAs.

Further details on the analyses of EGFR, KRAS, ALK, MSI, and miRNA-PCR are in Additional file 1.

## Results

### Clinicopathological characteristics

The occurrence of PEAC in the proband and his sister was associated with similar clinicopathological features, summarized in Table 1, such as comparable age at diagnosis, smoking history, and identical localization of the tumor. Colonoscopy in both cases revealed multiple benign polyps and diverticulosis of the sigmoid colon. Notably, the proband’s brother had also comparable age and smoking history, but the dramatic history of his disease precluded additional analyses.

### Histology, genetics, and miRNA profiling

**Fig. 1:**
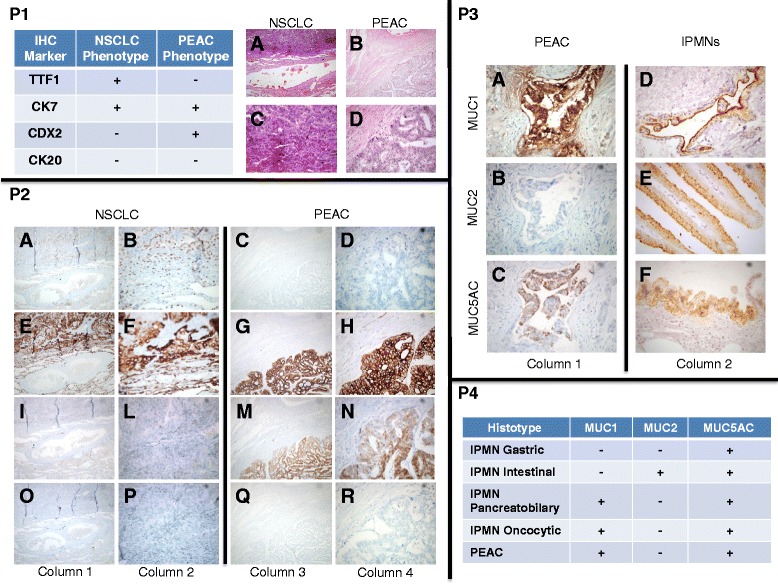
**P1** Histological analyses (hematoxylin and eosin) revealed the presence of two different phenotypes in the lung lesion of the proband. We found non-small cell lung cancer (NSCLC, 30 %; *A* magnification ×10; *B* magnification ×40) and pulmonary enteric adenocarcinoma (PEAC, 70 %; *C* magnification ×10; *D* magnification ×40). Table 1 summarizes the marker expression associated with these two different phenotypes. The expression of TTF1 and CDX2 was associated with NSCLC and PEAC, respectively. Strong expression of CK7 was observed in both phenotypes (100 %). **P2** Immunohistochemical signatures in NSCLC (*left panels*) and PEAC (*right panels*). TTF1 (*A*, *B*, *C,* and *D*); CK7 (*E* ***,*** *F* ***,*** *G*, and *H*); CDX2 (*I* ***,*** *L* ***,*** *M,* and *N*); and CK20 (*O* ***,*** *P* ***,*** *Q*, and *R*) staining showing co-localizations of TTF1/CK7 and CDX2/CK7 in NSCLC and PEAC, respectively. *Columns 1* and *3* (magnification ×10); *columns 2* and *4* (magnification ×40). **P3** Mucins (*MUCs*) expression in PEAC and pancreatic intraductal papillary mucinous neoplasms (*IPMNs*). Strong coexpression of MUC1 and MUC5AC was observed in the area with PEAC histology. Conversely, in the same area, we did no detect MUC2 expression. Examples of the positive expression of MUC1 (*D*), MUC2 (*E*), and MUC5AC (*F*) associated with different subtypes of IPMNs. *Columns 1* and *2* (magnification ×40). **P4** Summary of mucins (MUCs) expression in the different subtypes of IPMNs and in our PEAC samples showing similar molecular markers expression. In particular, the negative expression for MUC6 (data not shown) suggests a pancreatobiliary differentiation of areas within our PEAC sample

Macroscopically, a well-circumscribed lung nodule with a maximum diameter of 3.5 cm was found in both cases. Microscopically, the nodule was composed of medium-to-large complex glands, and tumor cells were cuboidal to tall columnar with a brush border and eosinophilic cytoplasm. Areas of irregular necrosis were present, as previously described [2]. H&E revealed two different phenotypes in the lung lesion of the proband: NSCLC (30 %) and PEAC (70 %; Fig. 1). Expression of TTF-1 and CDX2 are associated with NSCLC and PEAC, respectively. Similar results were observed in the cytological specimens of the proband’s brother (Additional file 2: Figure S1). Moreover, we observed strong coexpression of MUC1 and MUC5AC in the areas with PEAC histology, while in the same areas, we did not detect MUC2. In particular, the panel 4 of the Fig. 1 summarizes the MUCs expression compared to the subtypes of pancreatic intraductal papillary mucinous neoplasms (IPMNs). Considering the intestinal-type pulmonary histology and multiple colonic polyps, we evaluated on the tissue of the proband the MSI markers, which suggested the absence of microsatellite instability (MSS phenotype). KRAS (Gly12Asp) mutations but no EGFR/ALK aberrations were found.

**Fig. 2:**
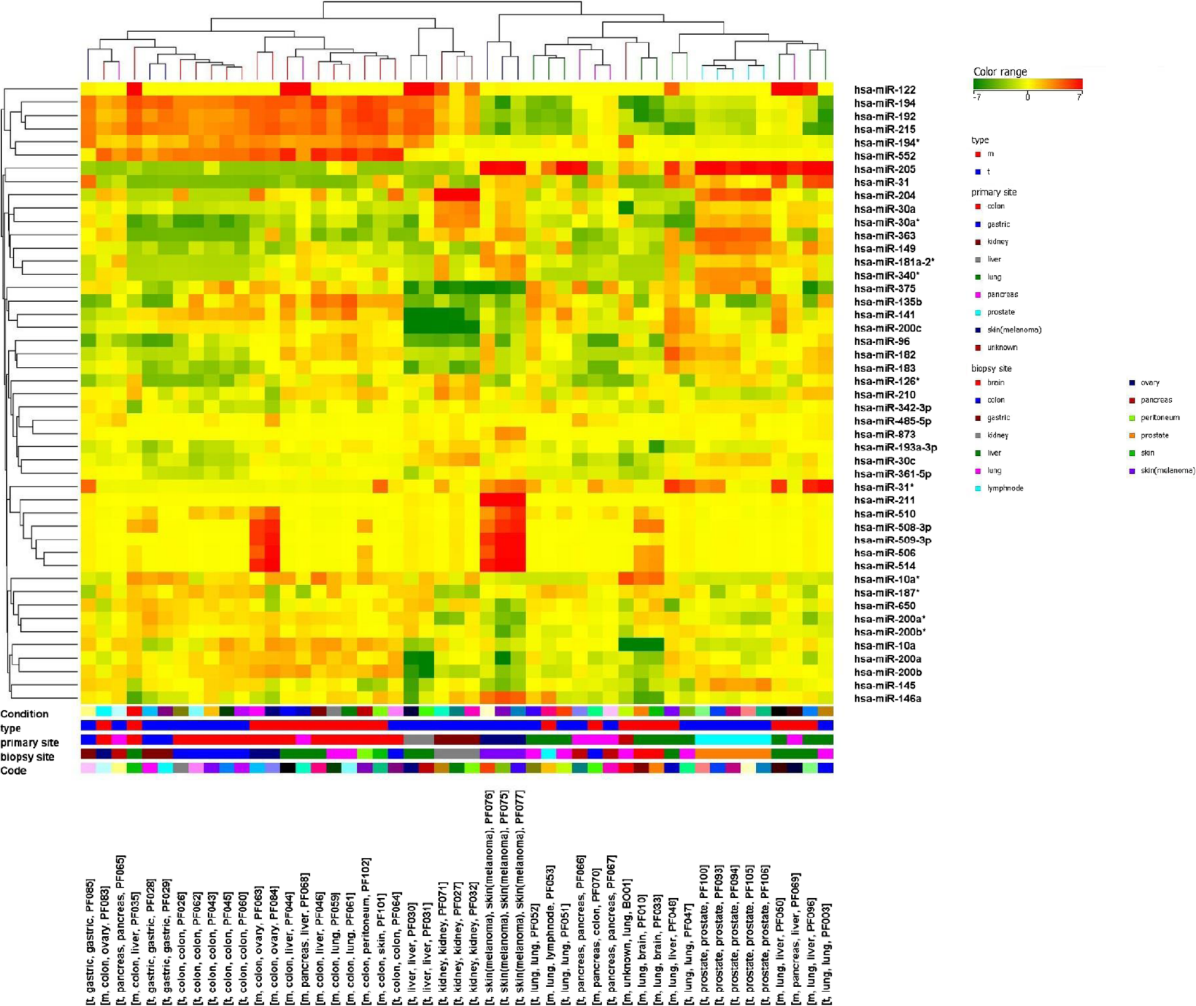
Unsupervised cluster analysis of 47 miRNAs in the proband’s sample of PEAC (“sample BO01”) compared to primary and metastatic tumors of lung cancer, CRC and PDAC, and also primary tumors of gastric, kidney, prostate, skin, and liver cancer. The colors represented on the heat-map correspond to the normalized miRNA expression value: *green* indicates downregulation while *red* indicates upregulation referred to the miRNA average value across all samples

The molecular characteristics of the proband’s PEAC were further evaluated using a miRNA profiling panel to predict tumor origin. Figure 2 shows the heat map representation of unsupervised clustering of 47 miRNAs in the proband’s sample (“sample BO01”) compared to primary and metastatic tumors of NSCLC, CRC and pancreatic ductal adenocarcinoma (PDAC), and primary gastric, kidney, prostate, skin, and liver cancers, as described previously [15]. This profiling revealed striking similarities with NSCLC (75.98 %) and a partial overlap with PDAC (23.34 %), but not with CRC (<0.5 %). The microarray data were confirmed by PCR analyses, focusing on five miRNAs, which showed a similar expression pattern in the proband’s brother (Additional file 3: Figure S2).

## Discussion

To our knowledge, this is the first study describing PEACs in members of the same family, associated with clinicopathological characteristics, histological, and genetic findings. Recently, El Hammoumi et al. described a patient with PEAC localized in the right lower lobe and associated with primary CRC [13]. However, miRNA profiling of the proband’s PEAC resembled that of lung cancer, with partial overlap of PDAC, but not CRC. This might at least in part explain the shorter disease-free survival (DFS) in the proband and his sister (1.5 and 12 months, respectively) compared to radically resected NSCLC patients, such as the 171 stage IA–IIIA NSCLC patients evaluated by Park et al., with a median DFS of 20 months [16].

Notably, we observed two different phenotypes (NSCLC and PEAC) in the lung lesions of the proband, which differed in IHC staining for TTF-1 and CDX2 markers. The NSCLC phenotype was characterized by TTF-1 positivity and CDX2 negativity. Conversely, the PEAC phenotype did not express the “lung cancer marker” TTF-1 but was instead characterized by the “intestinal marker” CDX2. Similar findings were observed in the proband’s sister and brother, underlying the potential role of these two markers (TTF-1, CDX2). Conversely, the proband and his sister showed different immunostaining for CK7 and CK20, commonly utilized to separate colorectal from other primary adenocarcinomas, especially the lung [12]. However, a previous description of 15 PEACs reported positivity for CK7 and CK20 in 93 and 27 % of cases, respectively [6].

Genetic analyses revealed KRAS (Gly12Asp) mutations, while no EGFR mutations or ALK rearrangements were described, in agreement with previous studies [2–13].

Using a 47-miRNA diagnostic array, we found that the miRNA profile of the proband’s PEAC was similar to that of lung carcinomas, but not CRC. However, we found a partial overlapping miRNA profile with PDAC, sharing some key miRNAs associated with tumor aggressiveness (miR-31*/-126*/-506/-508-3p/-514). In particular, miR-126*, mir-506, mir-508-3p, and mir-514 act as tumor suppressor genes, and their downregulation has been associated with proliferation, invasiveness, and poor survival outcomes [17–22]. Furthermore, miR-31* acts as oncogene and its’ overexpression was an independent prognostic for shorter survival [23]. The MUCs expression in our PEACs compared to the different subtypes of IPMNs suggest pancreatobiliary differentiation and again, not intestinal, despite histological similarity [24].

Qureshi et al. [8] described a patient affected by metastatic PEAC, showing good response after 4 cycles with carboplatin and pemetrexed. The same treatment was associated with disease progression in our patient, while his sister obtained disease stability after 6 cycles, suggesting that further studies are needed to define if the combination of platinum-based drugs and pemetrexed can be used as an option for first-line treatment in PEAC. Conversely, we do not recommend the CRC regimens, since we observed disease progression after XELOX, and the miRNA profiling showed no concordance with CRC. Finally, we observed disease stabilization after docetaxel, which is interesting, as taxanes showed efficacy in both NSCLC and PDAC. On the basis of these observations, as well as on the shorter DFS and miRNA profiling, we presume that PEAC represents an aggressive histological subtype of lung adenocarcinoma. Therefore, building upon previous evidence on the key role of histology and genetics to improve the management of advanced NSCLC, especially lung adenocarcinoma, we hypothesize that a better molecular understanding of PEAC will boost future therapeutic options.

### Ethics and consent

The research reported in the manuscript was performed with the approval of the appropriate ethics committee. Written informed consent was obtained for the molecular analyses and publication of this study.

## Additional files

Additional file 1:
**Analyses of EGFR, KRAS, ALK and MSI.** Detailed methods used for the analyses of EGFR, KRAS mutation, ALK rearrangement, MSI, and miRNA-PCR. (DOC 108 kb) Additional file 2: Supplemental Figure S1. Immunohistochemical analyses in the cytological sample of the proband’s brother. The stainings for TTF1, CK7, CDX-2, and CK20, summarized in the table, showed patterns of protein expression similar to the expression levels observed in the PEAC component of the proband’s sample. Left column (original magnification ×20); right column (original magnification ×40). (PPTX 32,747 kb) Additional file 3: Supplemental Figure S2. PCR analysis of miR-31*/-126*/-506/-508-3p/-514. Quantitative “miRNA-targeted” real-time PCR of five miRNA aberrantly expressed in the proband’s PEAC according to the microarray results showed that the microarray data were validated by PCR data as well as that the pattern of modulation of expression of these miRNA was similar in the proband’s brother. Columns and bars, average levels ± SD, compared using the 2^-ΔΔCt^ method to a pool of normal lung tissues, dashed line. (PPTX 158 kb)

## Abbreviations

ALK: anaplastic lymphoma kinase
CDX2: caudal-related homeobox 2
CK: cytokeratins
CRC: colorectal cancer
CT: computed tomography
DFS: disease-free survival
EGFR: epidermal growth factor receptor
FFPE: formalin-fixed, paraffin-embedded
H&E: hematoxylin & eosin
IHC: immunohistochemistry
IPMN: intraductal papillary mucinous neoplasm
KRAS: Kirsten rat sarcoma viral oncogene homolog
miRNA: microRNA
MSI: microsatellite instability
MSS: microsatellite stability
NEG: negative
NSCLC: non-small cell lung cancer
PDAC: pancreatic ductal adenocarcinoma
PEAC: primary pulmonary enteric adenocarcinoma
PET: positron emission tomography
POS: positive
TTF-1: thyroid transcription factor
