# Supplementary material for: MicroRNA profiling of primary pulmonary enteric adenocarcinoma in members from the same family reveals some similarities to pancreatic adenocarcinoma—a step towards personalized therapy
Source: Clin Epigenetics. 2015 Dec 16;7:129. doi: 10.1186/s13148-015-0162-5 (PMC4681170; doi:10.1186/s13148-015-0162-5)
Supplement: Additional file 1: — Analyses of EGFR, KRAS, ALK and MSI. Detailed methods used for the analyses of EGFR, KRAS mutation, ALK rearrangement, MSI, and miRNA-PCR. (DOC 108 kb) [file 13148_2015_162_MOESM1_ESM.docx]

**Supplemental data**

**Supplemental methods.**

**Analyses of EGFR, KRAS mutation, ALK rearrangement, and MSI.**

DNA and RNA were isolated with the QIAmp DNA extraction kit (Qiagen, S.p.A., Milan, Italy) and Ambion's RecoverAll™ Total Nucleic Acid Isolation Kit (Life Technologies, Carlsbad, CA) respectively, as described previously [Suppl. Ref#1].

Molecular analysis of the EGFR gene was performed by real-time PCR with the FDA approved TheraScreen EGFR RGQ PCR Kit (Qiagen) for research of 29 mutations in exons 18, 19, 20, and 21. The sensitivity of this test allows to determine up to 5% mutated DNA on a background of wild type genomic DNA. Molecular analysis of KRAS gene was performed by direct sequencing to search the KRAS mutations in exon 2, codon 12 and 13. The sensitivity of this test can determine up to 20-25% of mutated DNA on a background of wild type genomic DNA. Sequencing reactions were run on the ABI PRISM-310 Genetic Analyser (Applied Biosystems, Foster City, CA). ALK rearrangement status (2p23) was studied by standard fluorescence in situ hybridization, using Vysis ALK Break Apart FISH Probe kit, Abbott Molecular, IL. Microsatellite instability (MSI) was studied by multiplex amplification and sequencing with the CE-IVD CC-MSI kit (AB-Analitica, Advanced-Biomedicine, Padova, Italy), evaluating the following markers: BAT25, BAT26, D2S123, D17S250, D5S346 (“Bethesda panel”), BAT40, NR21, NR24, D18S58 and TGF RII, TPOX and TH01 (internal control).

**Quantitative real-time PCR for the analysis of miR-31/-126/-506/-508-3p/-514.** Quantitative "miRNA-targeted" real-time PCR was used to confirm the deregulation of five key miRNAs observed in the previous microarray analysis, evaluating their expression in the tumor samples from the proband and his brother as well as in lung normal tissues. From 10 to 100 ng RNA was reverse transcribed and the resulting cDNA was amplified using the specific TaqMan® MicroRNA assays (Applied Biosystems) for miR-31/-126/-506/-508-3p/-514 and the housekeeping RNU6 (Assay ID, 478015_mir, 477888_mir, 478958_mir, 479356_mir; 478961_mir and n256287, respectively). The PCR reactions were performed in a 20 μl final volume, using the TaqMan Universal PCR Master Mix, No AmpErase UNG, in the 7500HT sequence detection system (Applied Biosystems), in accordance with the manufacturer’s instructions. Amplification data were normalized to the expression of RNU6, and quantification of relative expression compared to normal lung tissues was performed using the 2^-ΔΔCt^ method, as described previously [Suppl. Ref#2]. Specimens were amplified in duplicate with appropriate non-template controls, and data were collected only when the CV was less than 2%.

**Supplemental References**

1. Role of CYB5A in pancreatic cancer prognosis and autophagy modulation. Giovannetti E, Wang Q, Avan A, Funel N, Lagerweij T, Lee JH, Caretti V, van der Velde A, Boggi U, Wang Y, Vasile E, Peters GJ, Wurdinger T, Giaccone G. J Natl Cancer Inst. 2014;106(1):djt346.

2. García-Santisteban I, Peters GJ, Giovannetti E, Rodríguez JA. USP1 deubiquitinase: cellular functions, regulatory mechanisms and emerging potential as target in cancer therapy. Mol Cancer. 2013;12:91.
