# Supplementary material for: MicroRNA profiling of primary pulmonary enteric adenocarcinoma in members from the same family reveals some similarities to pancreatic adenocarcinoma—a step towards personalized therapy
Source: Clin Epigenetics. 2015 Dec 16;7:129. doi: 10.1186/s13148-015-0162-5 (PMC4681170; doi:10.1186/s13148-015-0162-5)
Supplement: Additional file 2: Supplemental Figure S1. — Immunohistochemical analyses in the cytological sample of the proband’s brother. The stainings for TTF1, CK7, CDX-2, and CK20, summarized in the table, showed patterns of protein expression similar to the expression levels observed in the PEAC component of the proband’s sample. Left column (original magnification ×20); right column (original magnification ×40). (PPTX 32,747 kb) [file 13148_2015_162_MOESM2_ESM.pptx]

## Slide 1
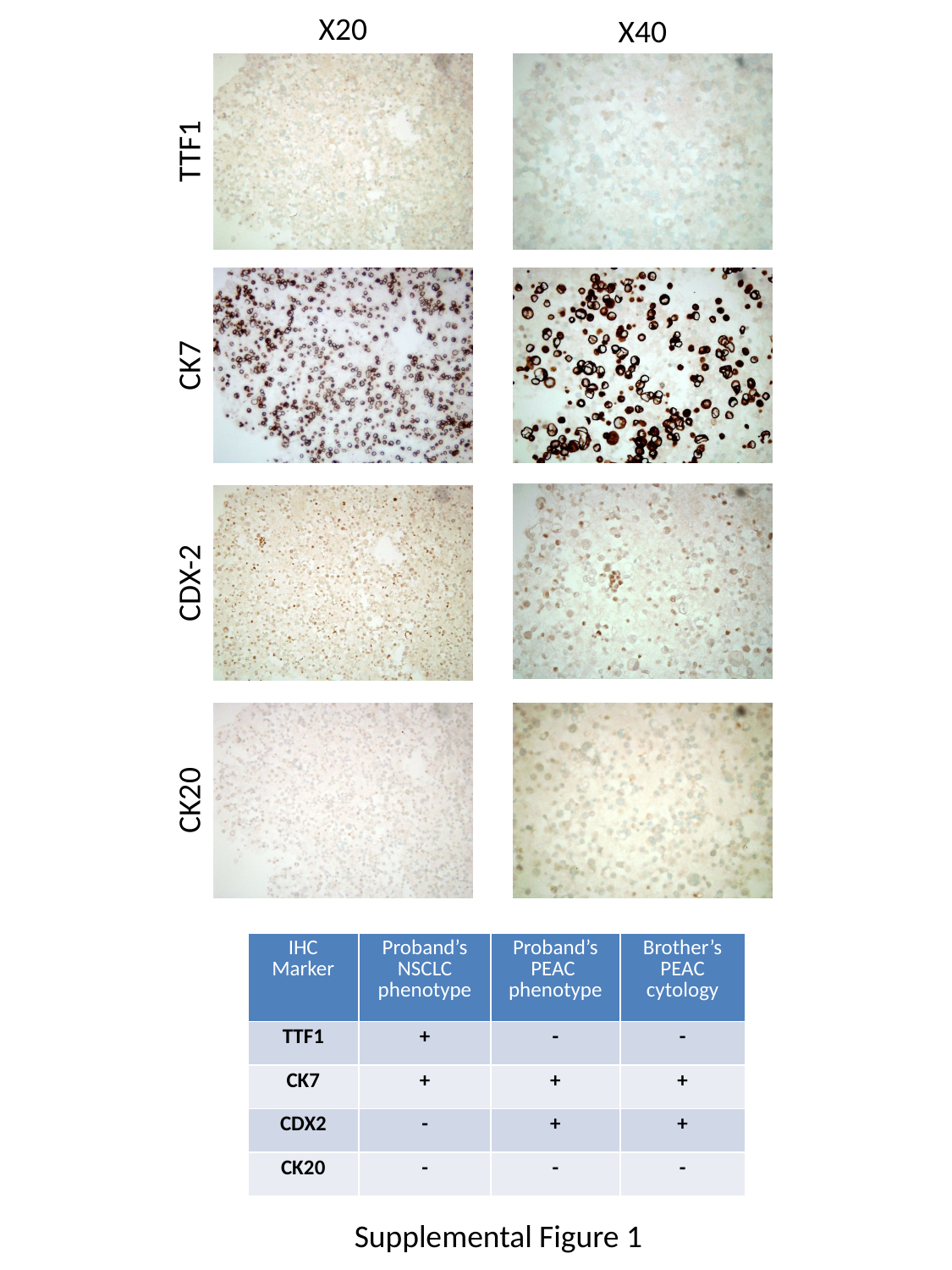

X20
X40
TTF1
CK7
CDX-2
CK20
| IHC Marker | Proband’s NSCLC phenotype | Proband’s PEAC phenotype | Brother’s PEAC cytology |
| --- | --- | --- | --- |
| TTF1 | + | - | - |
| CK7 | + | + | + |
| CDX2 | - | + | + |
| CK20 | - | - | - |
Supplemental Figure 1
