# Supplementary figures and images for: MicroRNA profiling of primary pulmonary enteric adenocarcinoma in members from the same family reveals some similarities to pancreatic adenocarcinoma—a step towards personalized therapy
Source: Clin Epigenetics. 2015 Dec 16;7:129. doi: 10.1186/s13148-015-0162-5 (PMC4681170; doi:10.1186/s13148-015-0162-5)

## Slide 1
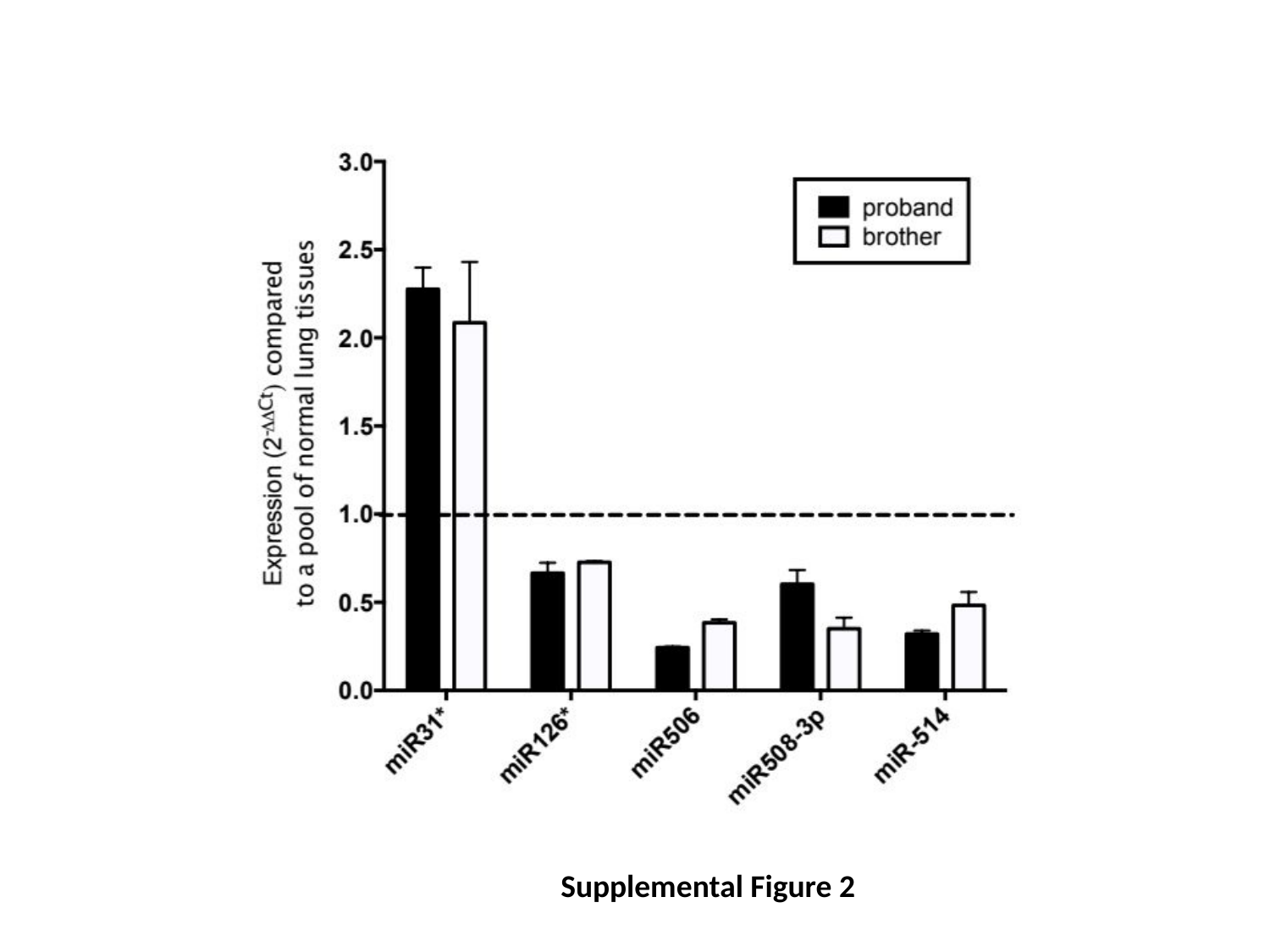

Supplemental Figure 2

Supplement: Additional file 3: Supplemental Figure S2. — PCR analysis of miR-31*/-126*/-506/-508-3p/-514. Quantitative “miRNA-targeted” real-time PCR of five miRNA aberrantly expressed in the proband’s PEAC according to the microarray results showed that the microarray data were validated by PCR data as well as that the pattern of modulation of expression of these miRNA was similar in the proband’s brother. Columns and bars, average levels ± SD, compared using the 2-ΔΔCt method to a pool of normal lung tissues, dashed line. (PPTX 158 kb) [file 13148_2015_162_MOESM3_ESM.pptx]
